# Detecting signs of retinal leakage in exudative AMD using Cirrus OCT versus SL SCAN-1, a novel integrated FD-OCT into a common slit lamp

**DOI:** 10.1007/s00417-015-2997-z

**Published:** 2015-04-23

**Authors:** M. Stehouwer, F. D. Verbraak, R. O. Schlingemann, T. G. van Leeuwen

**Affiliations:** Department of Ophthalmology, Academic Medical Centre, University of Amsterdam, Meibergdreef 9, 1105 AZ, Amsterdam, The Netherlands; Department of Biomedical Engineering and Physics, Academic Medical Centre, University of Amsterdam, Meibergdreef 9, 1105 AZ, Amsterdam, The Netherlands

**Keywords:** SL SCAN-1, Optical coherence tomography, Integrated SD-OCT into a slit lamp, Age-related macular degeneration, Exudative retinal disease

## Abstract

**Purpose:**

The purpose is to evaluate the interdevice and interobserver agreements between the SL SCAN-1 (a FD-OCT integrated into a common slit lamp) and a standard stand-alone FD-OCT device (the Cirrus) with regard to the presence or absence of signs of leakage in the retina in patients with exudative AMD and treated with anti-VEGF.

**Methods:**

Fifty-six patients, known to have exudative AMD and under treatment with anti-VEGF agents, were included. During a regular follow-up, OCT scans were made with the Cirrus (macular-cube pattern) and the SL SCAN-1 (radial-scan pattern). All scans were graded by two medical retina specialists for signs of intraretinal cysts, subretinal fluid accumulation, and thickening of the neurosensory retina. Presence of signs of leakage was concluded if one or more of the three signs were present.

**Results:**

In 91 % of the patients, the observers made identical conclusions for both devices of the presence of signs of leakage, resulting in an interdevice Kappa coefficient of 0.87. For the scans with disagreement about the presence or absence of signs of leakage, positive and negative conclusions were equally distributed between both devices, and differences were restricted to more subtle signs of leakage.

**Conclusion:**

The interdevice Kappa coefficient of 0.87 shows a high agreement between the SL SCAN-1 and the Cirrus in grading signs of leakage in exudative AMD. OCT images play a pivotal role in the diagnosis and management of exudative diseases like AMD, and the SL SCAN-1 provides a very efficient approach to these patients with the integration of the FD-OCT device into a common slit lamp.

## Introduction

Optical Coherence Tomography (OCT) is a non-invasive, non-contact imaging technique, providing images with detailed information of different structures of the eye. OCT has quickly evolved into a fast versatile imaging method, routinely used in the ophthalmic clinic. Currently, several Fourier Domain (FD)-OCT systems are commercially available. An alternative to these stand-alone systems is an FD-OCT-device integrated into a slit lamp. This SL SCAN-1 is an OCT device integrated into a common slit lamp with the ability to make OCT images of the anterior and posterior segments [1]. With the flexibility of the slit lamp, one can make OCT-scans of the observed area of interest during slit lamp biomicroscopy. The OCT scans are shown directly on a computer screen for interpretation. The OCT scans of the posterior segment can be made through a handheld lens, while the alignment for the sample arm is corrected by a fast Z-axis tracking system. With the use of a handheld lens, the part of the retina of which OCT sans can be made is identical to the field of view of the handheld lens. Basically, “what one can see is what one can scan.” With a handheld lens or a 3-mirror-contact lens even the far peripheral retina can be scanned, which has an added diagnostic value, for example, in the differentiation between senile retinoschisis and retinal detachment [2].

Next to the handheld lens, a specifically designed fixed lens (the fundus viewer) can be used to make scans of the posterior pole. By reducing the slit lamp beam to a small central light spot, this spot can be used as a central fixation point. Although this precludes the simultaneous view of the posterior pole, the combination of the fundus viewer with the central fixation spot ensures that one can easily make reliable and repeatable scans around the fixation point of the eye.

Nowadays, the OCT has a crucial role in the evaluation of patients with exudative retinopathies such as diabetic retinopathy and age-related macular degeneration (AMD). Often based on the presence or absence of signs of active leakage on OCT images, the decision is made to (re-)treat or to defer treatment with anti-VEGF injections [3].

This study evaluated the interdevice and interobserver agreement between the SL SCAN-1 and a standard stand-alone FD-OCT device, the Cirrus HD-OCT 4000 (Zeiss), with regard to the presence or absence of signs of leakage in the retina in patients with exudative AMD and treated with anti-VEGF.

## Patients and methods

Fifty-eight patients with exudative AMD were invited for this study. Two patients were excluded because of insufficient quality of the OCT scans to be used for analysis due to cataract. All 56 included patients were treated with anti-VEGF at the eye-hospital Zonnestraal, Hilversum, the Netherlands, and they were examined during a regular follow-up visit in the course of their treatment, between March and May 2011. All patients had received at least one previous series of intraocular injections, and presented with more subtle signs of leakage, instead of the more pronounced presence of signs of leakage at the time of their first treatment.

The study followed the tenets of the Declaration of Helsinki, and all patients gave their informed consent. All patients received mydriatic eyedrops in the examined eye (tropicamide and phenylephrine) and OCT scans were made by two experienced examiners, one using the SL SCAN-1 (Topcon) and one using the Cirrus HD-OCT 4000 (Zeiss). Both examiners were masked for the scans of the other examiner.

The SL SCAN-1 is a Fourier Domain Optical Coherence Tomography (FD-OCT) system with a broadband superluminescent diode (SLD) light source (bandwidth Δλ = 30 nm, central wavelength 840 nm), resulting in a axial resolution of 8 μm to 9 μm in tissue. The scan speed is 5000 A-scans per second and each B-scan is composed of 512 A-scans. Although scans can be made through a handheld lens, in this study scans were made through the fundus viewer lens designed by Topcon, a 60D Volk lens fixed in a lens holder. The lens is slightly tilted with respect to the optical path of the scanning beam to avoid reflexes of the lens surface. The stand has a detachable connection with the compatible slit lamps (Topcon model SL-D2/D4/D7/D8Z, Haag-Streit 900 series) and is positioned in the central channel of the common axis of the slit lamp. The working distance of the lens is approximately 13 mm, and can be adjusted with a knob on the side of the stand supporting a range of +5 to −15 diopter. With the fixed lens, one can use the light beam of the slit lamp, reduced to a small spot, as a central fixation light for the patient, parallel aligned to the OCT scanning beam. By using the slit lamp light as a fixation spot, the simultaneous view on the retina is precluded. In the present study, six line scans (with a length of 6 mm) were made with the SL SCAN-1 in a radial-scan pattern oriented 30 ° from one another around the fixation point. The Cirrus OCT scans were made using the Macular Cube 512x128 pattern, covering an area of 6x6 mm, with the patient looking to the fixation point. The Cirrus has a SLD light source with a central wavelength of 840 nm, resulting in a axial resolution of 5 μm in tissue. The scan speed is 27,000 A-scans per second and each B-scan is composed of 512 A-scans.

All OCT scans of this study were of sufficient image quality. The Cirrus uses the Signal Strength (SS) with a discrete scale of 1 to 10. Previous studies have shown a value of 6 or more to deliver images of sufficient quality to be reliably used in the clinic. The SLSCAN-1 uses a Quality Factor, from 0 to 100, and a value of 60 as the cutoff value for reliable clinical use is recommended.

Two observers, medical retina specialists experienced in the treatment of exudative AMD and reading of OCT images, graded in a random order all OCT scans independently. Both observers were masked for each other’s grades, for the course of the patients' AMD, and their response to treatment.

The scans were graded for signs of active leakage, presence or absence of intraretinal cysts (IRC), of subretinal fluid accumulation (SRF), or thickening of the neurosensory retina (Neuro Retinal Thickness, NRT). The NRT was estimated by the observers and not based on topographic maps. If one or more of these three signs was present, the OCT scan was graded as presence of signs of leakage.

The Cohen’s Kappa coefficients, most accurate for a dichotomous variable, were calculated to assess the interdevice and the interobserver agreements. After calculating the interdevice Cohen’s kappa coefficient per observer, an overall kappa coefficient was calculated by averaging. Similar analysis was performed to obtain the interobserver agreement. Commercial software (SPSS, version 16) was used for analysis.

## Results

**Fig. 1:**
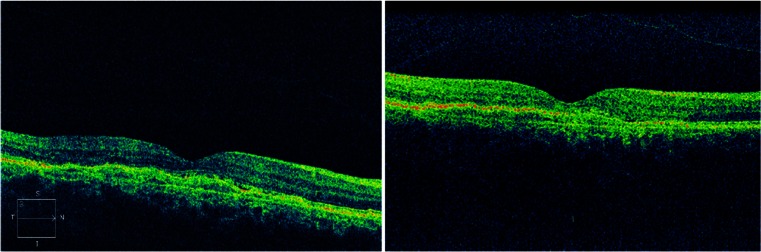
Both OCT scans (left Cirrus, right SL SCAN-1), made in the same eye of a patient, are of sufficient quality for grading (Cirrus SS > 6, SL SCAN-1 QF > 60)

**Fig. 2:**
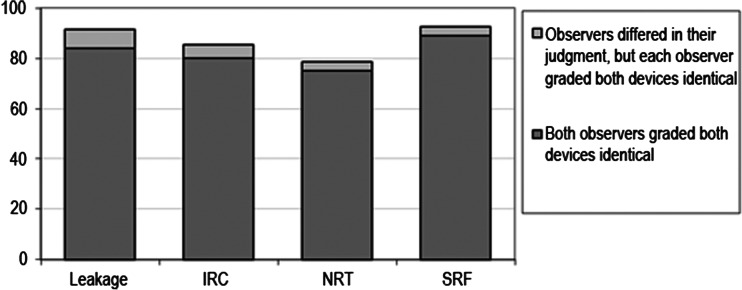
Percentage agreement between devices; percentage of OCT scans receiving identical conclusions of the observers per sign and per patient. For example: in 84 % both observers concluded identically for the presence or absence of signs of leakage based on scans of both devices, and in 7.3 %, both observers differed in their judgment of presence or absence of signs of leakage, but each observer graded the OCT scan on both devices to be equal

**Fig. 3:**
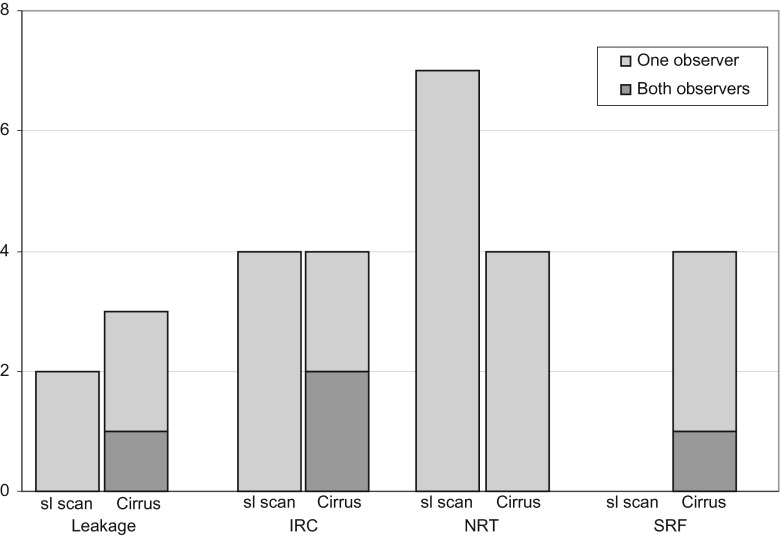
Comparison between the devices of discordant OCT scans, favouring positive findings, meaning the presence of a certain sign. Absolute number of disagreements in observations between the devices, favouring the positive findings per device. This includes all the OCT scans of the patients graded differently on the two devices, not related to the observer. For example: presence or absence of signs of leakage: both observers concluded that in one patient signs of leakage were present on the OCT scan made with the Cirrus and not on the scans made with the SL SCAN-1 (black). In four patients (grey), only one of the observers differed in his grading between scans made with the two devices (in two patients, signs of leakage was seen only on the SL SCAN-1, and in another two patients only on the Cirrus)

Fifty-six patients diagnosed with exudative AMD and treated with anti-VEGF were included in this study. The mean age of all subjects was 80 years (range of 55–96 years), and 37 of them were female. OCT scans could be made in all 56 eyes with both the SL SCAN-1 and the Cirrus; all scans were of sufficient quality for grading (Cirrus SS > 6, SL SCAN-1 QF > 60). An example of the OCT scans made with both devices in one patient is shown in Fig. 1. In 13 eyes out of the 56 patients, there was not any sign of leakage seen by both observers on both devices. The interdevice agreements are shown in Table 1. A Kappa coefficient of 0.87 was calculated for the presence or absence of signs of leakage, and the table shows a good to superior agreement between the devices for each individually graded sign. Figure 2 shows the percentage of OCT scans receiving identical grades per patient, with a unanimous 84 % for the presence or absence of signs of leakage. In 7.3 % (n = 4) a discrepancy in grading was present between observers, but each observer graded the OCT scan of both devices equal. Overall, in 91 % of the patients the observer's conclusion based on the SL SCAN-1 was identical to the one based on the Cirrus. Figure 3 shows the absolute number of disagreements between the devices, favouring the positive findings per device. This number consisted of patients where both observers graded differently with both devices, and patients where only one of the observers differed in his grading between the two devices. For example: for only one patient both observers concluded that there were signs of leakage on the OCT scan made with the Cirrus and not on the scans made with the SL SCAN-1. In four patients, only one of the observers differed in his grading between scans made with the two devices (in two patients signs of leakage were seen only on the SL SCAN-1, and in another two patients only on the Cirrus). Table 2 shows the Kappa coefficient for the interobserver agreements. For the outcome "signs of leakage" the kappa was 0.75, which can be interpreted as a substantial agreement. Discrepancy between observers in the outcome "signs of leakage" was found in nine patients. In four of these nine patients, the observer made the same conclusion based on the scans of both devices, but differed in his conclusion with the other observer. The outcome "presence of signs of leakage" was based on the presence of intraretinal cysts, and / or subretinal fluid accumulation and / or thickening of the neurosensory retina. Of these signs, the highest agreements (interobserver and interdevice) were found for subretinal fluid and the lowest for thickening of the neurosensory retina.

**Table 1:** Interdevice agreement

	Kappa coefficient	95 % CI
**Leakage** *	**0.87**	**0.80**–**0.94**
IRC	0.80	0.72–0.88
SRF	0.89	0.83–0.95
NRT	0.76	0.68–0.88

* Presence of signs of leakage based on the presence of one or more of the following signs: intraretinal cysts (*IRC*), subretinal fluid (*SRF*), or neurosensory retina thickening (*NRT*)

Kappa coefficient is an average of the interdevice kappa coefficient of each observer

**Table 2:** Interobserver agreement

	Kappa coefficient	95 % CI
**Leakage** *	**0.75**	**0.68**–**0.82**
IRC	0.76	0.70–0.83
SRF	0.84	0.79–0.90
NRT	0.67	0.60–0.75

* Presence of signs of leakage based on the presence of one or more of the following signs: intraretinal cysts (*IRC*), subretinal fluid (*SRF*), or neurosensory retina thickening (*NRT*)

Kappa coefficient is an average of the interobserver kappa coefficient of each device

## Discussion

This study shows that the OCT scans made with the SL SCAN-1, an OCT-device integrated into a slit lamp, have a comparable accuracy to detect signs of leakage in patients with exudative AMD as the scans made with the Cirrus, a representative commonly used stand-alone FD-OCT device. In 91 % of the patients, an identical conclusion was made for the presence or absence of signs of leakage based on the OCT scans of both devices by each observer, with a kappa of 0.87. This means that the scans of both devices are of a comparable quality to grade signs of leakage.

The outcome "signs of leakage" is based on one or more of the following signs: intraretinal cysts, subretinal fluid, and neurosensory retina thickening. Of these signs, SRF had the highest percentage of unanimous agreement between devices (see Fig. 2), which resulted in an interdevice agreement Kappa coefficient of 0.89 (Table 1). The interdevice agreement of neurosensory retina thickening was the lowest with a value of 0.76. An explanation for the variety in agreement between these two signs could be that subretinal fluid is, even when minimally present, a clear sign on an OCT scan. Thickening of the retina can be subtle and is more influenced by a subjective interpretation. In this study no actual measurements of the retinal thickness were made, and thickness was estimated by the observers. In clinical practice, retinal thickness measurements as provided by the segmentation software of any OCT device is often unreliable in patients with exudative AMD as a consequence of artefacts due to the complex structural changes in the outer retina. [4–6]. Consequently, clinicians need to estimate most of the time pathological increases in retinal thickness. As could be expected, the highest disagreement between the devices and the lowest kappa was found for the subjective interpretation of the sign neurosensory retinal thickening (Fig. 2, Table 2). In case one of the devices, Cirrus or SL SCAN-1, would have been more sensitive to detect subtle signs of leakage, one would expect in cases of disagreement between the devices, the most sensitive device to be more often positive for the presence of signs of leakage. In reality, as shown in Fig. 3, the disagreement occurred without preference for one or the other device.

There are important differences between the SL SCAN-1 and the Cirrus that should be taken into account. The Cirrus images were made with a cube scan of 128 scan lines with an axial resolution of 5 μm. The SL SCAN-1 was operated with a radial-scan pattern of six lines with an axial resolution of 8–9 μm. Consequently, the scans of the SL SCAN-1 are denser near the centre of the macula and much less dense at the edges of the scan pattern compared to the cube scan of the Cirrus. Signs of leakage not centrally located, therefore, could be missed with this setup, although this was not reflected in the results. The higher number of scan lines and the higher resolution of the scans made with the Cirrus were not clearly reflected in the results of this study.

Only “substantial” agreements were found for the interobserver Kappa coefficients. This study was performed in a clinical setting and experienced observers, specialists in treating exudative AMD, graded the OCT images. This study confirms that there is a rather large variation in the interpretation of scans in clinical practice, between different observers, as mention in other papers [7–9]. The observed variation is at the same order of magnitude as in these previous studies.

With the SL SCAN-1 a fixation light is offered to the patient by using the light beam of the slit lamp reduced to a small spot. This precludes the simultaneous view of the retina. Although a few patients had an eccentric fixation, all patients were asked to observe the central fixation light during imaging with both devices. There was no difference between the two devices in the position of the imaged area of the retina. However, using most standard OCT devices, it is possible to observe the macula before scanning and adjust the position of the scanned area in case of overt eccentric fixation; this is not possible with the SL SCAN-1 when the light beam is reduced to a small fixation spot.

A shortcoming of the present study is that the observers were not masked for the devices. The type of scan pattern immediately identifies the used device, and therefore, masking for the device was not possible.

In conclusion, the SL SCAN-1 enables viewing and scanning the retina simultaneously, providing a very efficient approach to the patient with a retinal disease. The OCT scan is central in the decision to (re)treat a patient with wAMD showing the presence or absence of signs of leakage. This study shows that, in almost all patients with wAMD, identical clinical decisions with regard to signs of leakage are made based on the OCT scans made with either the SL SCAN-1 or the Cirrus OCT.
